# Synergistic anticancer effects of ABT-199 and Vorinostat encapsulated in PLGA nanoparticles: Formulation, characterization, and antiproliferative effects against colorectal cancer cells

**DOI:** 10.1371/journal.pone.0334427

**Published:** 2025-10-10

**Authors:** Dina Raed Abu Alsamen, Zainab Zaki Zakaraya, Anas Abed, Alhareth A. Alsa’d, Maysoon Raed Alnajdawi

**Affiliations:** 1 Department of Pharmaceutical Sciences, Faculty of Pharmacy, Al-Ahliyya Amman University, Amman, Jordan; 2 Depatment of Pharmaceutical Chemistry, School of Pharmaceutical Sciences, University of Sains Malaysia, Pulau Pinang, Malaysia; BRAC University, BANGLADESH

## Abstract

The combination of ABT-199, a BCL-2 inhibitor, and Vorinostat, a histone deacetylase inhibitor, holds great potential in colorectal cancer therapy due to their synergistic effects. However, their poor solubility and bioavailability present challenges for effective treatment. This study aimed to co-encapsulate these drugs in poly(lactic-co-glycolic acid) nanoparticles to enhance stability, control drug release, and preserve their synergistic anti-proliferative effects in colorectal cancer cells. This study initially focused on evaluating the anti-proliferative activity of free ABT-199 and Vorinostat in HT-29 and HCT116 colorectal cancer cells. The drugs demonstrated potent cytotoxic effects, with Vorinostat exhibiting IC_50_ values of 1.32 µM in HT-29 cells and 2.04 µM in HCT116 cells, while ABT-199 displayed IC_50_ values of 4.04 µM and 5.49 µM, respectively. To investigate the interaction between ABT-199 and Vorinostat, the combination index was calculated using the Chou-Talalay method. The analysis revealed strong synergism between the drugs in both cell lines, with CI values consistently below 1 across all tested molar ratios. The most pronounced synergy was observed at a 1:1 molar ratio, which exhibited the lowest CI values. Building on these results, ABT-199-loaded nanoparticles (ABT-NPs), Vorinostat-loaded nanoparticles (VOR-NPs), and dual-loaded nanoparticles (DLNPs) were formulated using the nanoprecipitation method. ABT-NPs and VOR-NPs had sizes of 210.6 ± 6.2 nm and 202.5 ± 5.6 nm, with encapsulation efficiencies of 73.2 ± 4.81% and 86.4 ± 5.5%, respectively. The DLNPs, which co-encapsulated both drugs at a 1:2 molar ratio, exhibited a size of 210 ± 7.3 nm and maintained good stability. Cytotoxicity studies revealed that both ABT-NPs and VOR-NPs retained comparable anti-proliferative effects to the free drugs, with IC_50_ values close to those of their unencapsulated counterparts. Furthermore, DLNPs enhanced the anti-proliferative effect, significantly increased the apoptotic cells as measured by flow cytometry which was coincided with an increasing caspase-3 activity in both HT-29 and HCT116 cells, indicating an enhanced apoptotic response.

## 1. Introduction

Cancer remains a major global health challenge, with both incidence and mortality rates steadily rising. In 2023, the United States alone recorded 1,958,310 new cancer cases and 609,820 cancer-related deaths, highlighting the growing burden of this disease [1]. Projections suggest that by 2030, cancer could claim up to 30 million lives annually worldwide [2]. Among all cancer types, colorectal cancer is particularly aggressive and frequently poses challenges in treatment. Standard therapies, including chemotherapy, often suffer from limitations such as non-selective cytotoxicity, poor pharmacokinetics, and significant adverse effects. Among the various therapeutic strategies explored, targeting the apoptotic pathway has shown considerable promise.

ABT-199, also known as Venetoclax, is a selective BCL-2 inhibitor that has demonstrated therapeutic potential in multiple cancers, including acute myeloid leukemia (AML) [3], relapsed chronic lymphocytic leukemia [4], hormone receptor-positive breast carcinoma [5] and cervical cancer [6]. Vorinostat (VOR), an FDA-approved histone deacetylase (HDAC) inhibitor, has demonstrated broad-spectrum anticancer activity through epigenetic modulation. it is approved for cutaneous T-cell lymphoma [7], and has been investigated for hematological malignancies. Additionally, Vorinostat has shown promise in solid tumors such as glioblastoma multiforme and non-small cell lung cancer [8]. Individually, both ABT-199 and Vorinostat demonstrated potent anti-proliferative effects in preclinical models. Their combination has garnered attention for its potential to enhance therapeutic efficacy through synergistic interactions. By utilizing complementary mechanisms of action- BCL-2 inhibition inducing intrinsic apoptosis, and HDAC inhibition reactivating silenced tumor suppressor genes- the combination may increase cancer cell sensitivity and reduce drug resistance, making them an attractive option for combination therapy.

However, despite their promise, both drugs face significant challenges that limit their therapeutic potential. ABT-199, while highly effective, is associated with serious systemic side effects, including an increased risk of infections, which may necessitate treatment interruptions or suboptimal dosage adjustments [9]. Additionally, ABT-199 suffers from a poor pharmacokinetic profile, including poor solubility and limited bioavailability. Similarly, the effectiveness of Vorinostat is hindered by poor aquoes solubility, low oral bioavailability, and rapid hepatic metabolism, which restrict its systemic exposure and clinical utility [10]. Moreover, due to the distinct pharmacokinetic profiles of both agents, achieving the desired synergistic ratio at the tumour site can be challenging.

These limitations highlight the need for innovative drug delivery strategies that can improve therapeutic outcomes while minimizing systemic toxicity.

Nanotechnology has emerged as a promising approach in cancer therapy, offering solutions to many of the above challenges. Nanoparticles (NPs) can enhance the pharmacokinetic properties of anticancer drugs, improve tumor targeting via the enhanced permeability and retention (EPR) effect, and reduce off-target toxicity [11]. Importanly, the co-encapsulation of of multiple drugs within a single nanoparticle system enables synchronized drug release, potentially leading to enhanced synergistic effect at the tumour site. Among the various nanocarriers, poly(lactic-co-glycolic acid) (PLGA) has gained substantial attention due to its favorable characteristics, including biocompatibility, biodegradability, and the capacity for controlled drug release [12]. PLGA nanoparticles have been extensively studied for delivering chemotherapeutics, as they are capable of encapsulating both hydrophilic and hydrophobic agents, thereby improving drug stability, prolonging tumor site retention, and reducing systemic side effects [13,14].

This study aimed to investigate the synergistic anticancer effects of ABT-199 and Vorinostat in colorectal cancer cells, both as free drugs and when encapsulated in PLGA nanoparticles. The objectives were first to quantify the anti-proliferative effects and drug synergy between ABT-199 and Vorinostat using the combination index (CI) method, and second, to develop and characterize dual-drug-loaded nanoparticles (DLNPs) and evaluate whether nanoparticle encapsulation could retain or enhance their therapeutic efficacy. By addressing both pharmacological synergy and nanoparticle-based drug delivery, this study proposes a potentially more effective therapeutic strategy for colorectal cancer, while overcoming the limitations associated with each agent.

## 2. Materials and methods

### 2.1. Nanoparticle formulation

Nanoparticles were synthesized in 20 mg batches using the nanoprecipitation technique with PLGA 502H (Sigma-Aldrich). The polymer was dissolved in 1 mL of acetone to form the organic phase, which was then added dropwise to 7 mL of distilled water (aqueous phase) under continuous stirring. For drug-loaded NPs, 1 mg of ABT-199, Vorinostat, or a combination of both was dissolved in 100 μL of DMSO and then incorporated into the organic phase to ensure uniform drug distribution within the PLGA matrix. The resulting suspension was stirred overnight to ensure complete evaporation of acetone.

Nanoparticle purification was performed through three wash and centrifugation cycles at 16,000g for 15 minutes each. During each cycle, the supernatant containing residual DMSO and unencapsulated drugs was discarded, and the nanoparticle pellet was resuspended in fresh PBS; this process effectively removes DMSO due to its high water solubility. To confirm the absence of residual DMSO, Fourier Transform Infrared (FTIR) spectroscopy was performed on dried dual-loaded nanoparticles (DLNPs). The measurements were conducted at ambient room temperature, utilizing the transmission mode of operation using Lambda Scientific FTIR model 7600 coupled with attenuated total reflection ZnSe crystal. The resolution for these measurements was set at 8 cm^−1^ and 32 cm^−1^, scanning the range between (400–4000) cm^−1^. The DLNP spectrum showed no detectable DMSO peaks, confirming effective removal, while pure DMSO exhibited characteristic peaks at ~1050 cm ⁻ ¹ and ~700 cm ⁻ ¹ (S1 Fig).

The purified NPs were then resuspended in PBS for further characterization and cellular experiments.

To assess nanoparticle uptake, the polymer was modified by blending 1 mg of PLGA-Rhodamine B (50:50 lactide:glycolide) (Sigma-Aldrich) with 19 mg of PLGA 502H. These fluorescently labeled NPs were prepared using the same nanoprecipitation method described in this section.

### 2.2. Nanoparticle characterizations

The hydrodynamic size, polydispersity index (PDI), and zeta potential of the NPs were measured using a Malvern Zetasizer (Malvern Instruments, UK). The NPs were resuspended in PBS at a final concentration of 0.2 mg/mL, and the measurements were performed by transferring the suspension into a cuvette for analysis. To assess the colloidal of the nanoparticles, samles were stored at different condictions: 4°C, room temperature, and 37° for a period of 30 days. At predetermined time points, samples were withdrawn and analyzed for changes in particle size, PDI, and zeta potential.

For morphological analysis, transmission electron microscopy (TEM) was performed. A drop of freshly prepared nanoparticle suspension was placed onto a carbon-coated copper grid using a micropipette. The sample was allowed to settle for 2–3 minutes to promote adsorption of nanoparticles onto the grid surface. Subsequently, the grid was negatively stained by adding a drop of 2% (w/v) phosphotungstic acid (PTA), adjusted to pH 6.8, to enhance contrast. After gentle blotting and air drying, the samples were imaged using a TEM operating at an accelerating voltage of 80–120 kV.

### 2.3. Quantification of drug loading within the PLGA nanoparticles

The drug content within the PLGA nanoparticles was determined by dissolving the nanoparticles pellet in a 1:1 mixture of acetonitrile and DMSO, which facilitated the release of encapsulated drugs. ABT-199 content was quantified spectrophotometrically by measuring the absorbance at 420 nm using the BioTek Cytation 5 multi-mode plate reader. Drug concentrations were calculated based on a standard curve constructed from known concentrations of free ABT-199.

For Vorinostat, quantification was performed using high-performance liquid chromatography (HPLC) with detection at 241 nm under isocratic conditions. Separation was acheived using a C18 reverse-phase column (Phenomenex, 100x4.5 mm, 5 μm) with a mobile phase of acetonitrile and 0.1% formic acid. The flow rate was set at 1 mL/min, and the column temperature was set to 30°C. A 10 μL sample was injected for each run.

The encapsulation efficiency (EE%) was calculated using Equation (1):


$$Encapsulation\;Efficiency\%=\frac{drug\;mass\;in\;the\;NPs}{drug\;mass\;added\;to\;formulation}\;x\;\text{1}\text{0}\text{0}\%$$ (1)

### 2.4. *In vitro* drug release

To evaluate the drug release profile from the nanoparticle formulations, high-retention seamless cellulose dialysis tubing (Sigma-Aldrich) was employed. A total of 20 mg of drug-loaded nanoparticles was suspended in PBS and placed inside the dialysis tubing. The dialysis tubing was then submerged in a release medium consisting of of PBS supplemented with 10% (v/v) fetal bovine serum (FBS) and 1% (v/v) Tween-20, maintained at 37 ºC under magnetic stirring.

At specific time points, the residual nanoparticle suspension was collected by centrifugation at16,000 *g* for 15 minutes at 4 ºC. Later, the resulting nanoparticle pellet was dissolved in 1 mL of 1:1 (v/v) of acetonitrile and DMSO to release the remaining encapsulated drug. Drug content was quantified as previously described in section 2.3.

### 2.5. Cell culture

The colorectal cancer cell lines HCT116 (RRID: CVCL_0291) and HT-29 (RRID: CVCL_0320) were sourced from the American Type Culture Collection (ATCC, Manassas, VA, USA).

Cells were maintained in high-glucose Dulbecco’s Modified Eagle Medium (DMEM) (Euroclone) supplemented with 10% FBS (Euroclone), 1% Penicillin/Streptomycin solution (containing 5000 units/mL of both penicillin and streptomycin) (Gibco), and 1 mM sodium pyruvate (Euroclone). Cultures were incubated at 37°C in a humidified atmosphere containing 5% CO₂.

Cells were routinely tested for mycoplasma contamination using a mycoplasma detection kit (Invivogen).

### 2.6. Confocal microscopy

To assess the cellular uptake of the nanoparticles, flow cytometry and confocal microscopy were employed. For the flow cytometry, a total of 1 × 10⁵ cells were seeded into a 6-well plate and incubated overnight to allow for adherence. The following day, the culture medium was discarded, and the cells were treated with Rhodamine B-loaded nanoparticles at a concentration of 200 μg/mL for 6 hours. After incubation, the nanoparticle-containing medium was removed, and the cells were washed three times with PBS before being collected into a 15 mL Falcon tube. The cell suspension was centrifuged to obtain a pellet, which was then resuspended in 500 μL of PBS. The fluorescence intensity of the cells (PE channel) was measured using a BD Accuri C6 Plus flow cytometer. Appropriate gating was applied to exclude debris, and data were acquired for 10,000 gated events. Flow cytometry results were analyzed using FlowJo software to generate the final graphs.

For confocal microscopy. Cells were seeded at a density of 1 × 10⁵ cells per well onto an 8-well chamber slide (BD Falcon) and incubated overnight to facilitate adherence. The following day, the culture medium was removed, and cells were treated with 200 μg/mL Rhodamine B-loaded nanoparticles for 6 hours. At the designated time point, cells were washed with ice-cold PBS and subjected to acid stripping buffer for 5 minutes to remove surface-bound particles. After another PBS wash, cells were fixed with ice-cold 4% paraformaldehyde in PBS for 20 minutes. Subsequently, cells were washed three times with PBS and permeabilized using 0.5% Triton X-100 in PBS for 5 minutes. After an additional set of PBS washes (3x), Vectashield antifade mounting medium containing DAPI (Vector Laboratories) was applied, and coverslips were carefully mounted. Confocal imaging was performed using a Leica SP8 confocal microscope (Leica, UK), equipped with an HCX PL APO 63 × oil immersion objective (1.4–0.6 NA). Images were captured at a resolution of 1024 × 1024 pixels with a scan speed of 400 Hz. Fluorescence was detected following excitation with various lasers: UV diode (405 nm), argon (488 nm), DPSS (561 nm), and helium-neon lasers (543 nm, 594 nm, and 633 nm), depending on the fluorophore. Image analysis was conducted using Leica LAS X software (Leica, UK).

### 2.7. MTT viability assay

Cell viability was assessed using the MTT assay (Genchemn World) following the manufacturer’s protocol. HCT116 and HT-29 cells were seeded at a density of 5 × 10^3^ cells per well in 96-well plates and incubated overnight. The following day, cells were treated with either free drugs or nanoparticle formulations, and incubated for 72 hours. For nanoparticle treatments, the purified NPs were further diluted in high-glucose DMEM to achieve the desired drug concentrations before being applied to the cells. At the designated time point, 0.05 mg/mL of MTT reagent was added to each well, and the plates were incubated for 2–4 hours to allow the formation of formazan crystals. Following incubation, the media was carefully removed, and the formazan crystals were dissolved in 100 µL of DMSO to ensure complete solubilization. Absorbance was then measured at 570 nm using BioTek Cytation 5 multi-mode plate reader (BioTek). Cell viability was epressed as a percentage relative to untreated control cells. IC_50_ values, representing the drug concentration required to inhibit 50% of cell viability, were calculated by fitting the dose-response data (percentage viability vs. log[drug concentration]) to a non-linear regression model (variable slope, four-parameter logistic equation) using GraphPad Prism software.

### 2.8. Caspase-3 activity

HT-29 and HCT116 cells were seeded in 6-well plates and incubated overnight. The following day, cells were treated with 2 µM of ABT-NPs, 1 µM of VOR-NPs, or their nanoparticle combination for 72-hours. At the designated time-point, the cells were lysed using an optimized volume of ice-cold radio-immunoprecipitation (RIPA) buffer supplemented with protease inhibitor cocktail (Roche, UK). Proten concentration in the resulting lysates was quantified using the Bicinchoninic acid (BCA) assay (Pierce, UK).

Caspase-3 enzymatic activity was measured using the calorimetric Caspase-3 assay kit (Abcam, UK). Briefly, 50 µg of protein was added to each well of a 96-well plate, followed by the addition of 50 µL of 2X reaction buffer (containing 100 mM of DDT) and 5 μL of 4 mM DEVD-p-NA substrate (200 μM final concentration). The mixture was gently mixed and incubated at 37°C for 60 minutes. Eventually, the absorbance was read at Optical Density of 400 nm using a Bio-Tek Cytation 5 multi-mode plate reader. Caspase-3 activity was expressed as fold change relative to untreated control.

### 2.9. Apoptosis detection via Annexin V/PI staining

HCT116 and HT-29 cells were seeded in 6-well plates at a density of 4 × 10⁵ cells per well and incubated overnight to allow for adherence. The following day, cells were treated with 1 µM Vorinostat-loaded nanoparticles (Vor-NPs) and 2 µM ABT-199-loaded nanoparticles (ABT-NPs) for a duration of 72 hours.

At the end of the treatment period, the culture medium containing floating (dead) cells was collected along with the adherent cells, which were gently detached and transferred into 15 mL Falcon tubes. The cell suspensions were centrifuged at 500 × g for 5 minutes to pellet the cells. The supernatant was discarded, and the cell pellet was resuspended in 300 μL of 1 × binding buffer (BD Biosciences), followed by the addition of 3 μL of Annexin V-FITC and 2 μL of Propidium Iodide (PI). Samples were incubated in the dark for 15 minutes at room temperature.

Stained cells were analyzed using a BD Accuri™ C6 Plus flow cytometer. Appropriate gating was applied to exclude debris while retaining both live and dead cells. our final gates were applied to differentiate between Annexin V/PI negative (live cells), PI-only positive (necrotic cells), Annexin V only positive (early apoptotic cells) and Annexin V/PI positive (late apoptotic cells).

### 2.10. Data analysis

Statistical analyses were conducted using GraphPad Prism 8.0 software. One-way ANOVA was employed to compare experimental results, with significance levels denoted as follows: * = p ≤ 0.05, ** = p ≤ 0.01, and *** = p ≤ 0.001. Experiments were replicated at least 2–3 times.

Combination indices (CI) were calculated using the Chou-Talalay method via CompuSyn software, where CI values of <1, = 1, and >1 indicate synergism, additivity, and antagonism, respectively.

## 3. Results

### 3.1. Assessment of the cellular response to ABT-199 and Vorinostat in colorectal cancer cells

**Fig 1:**
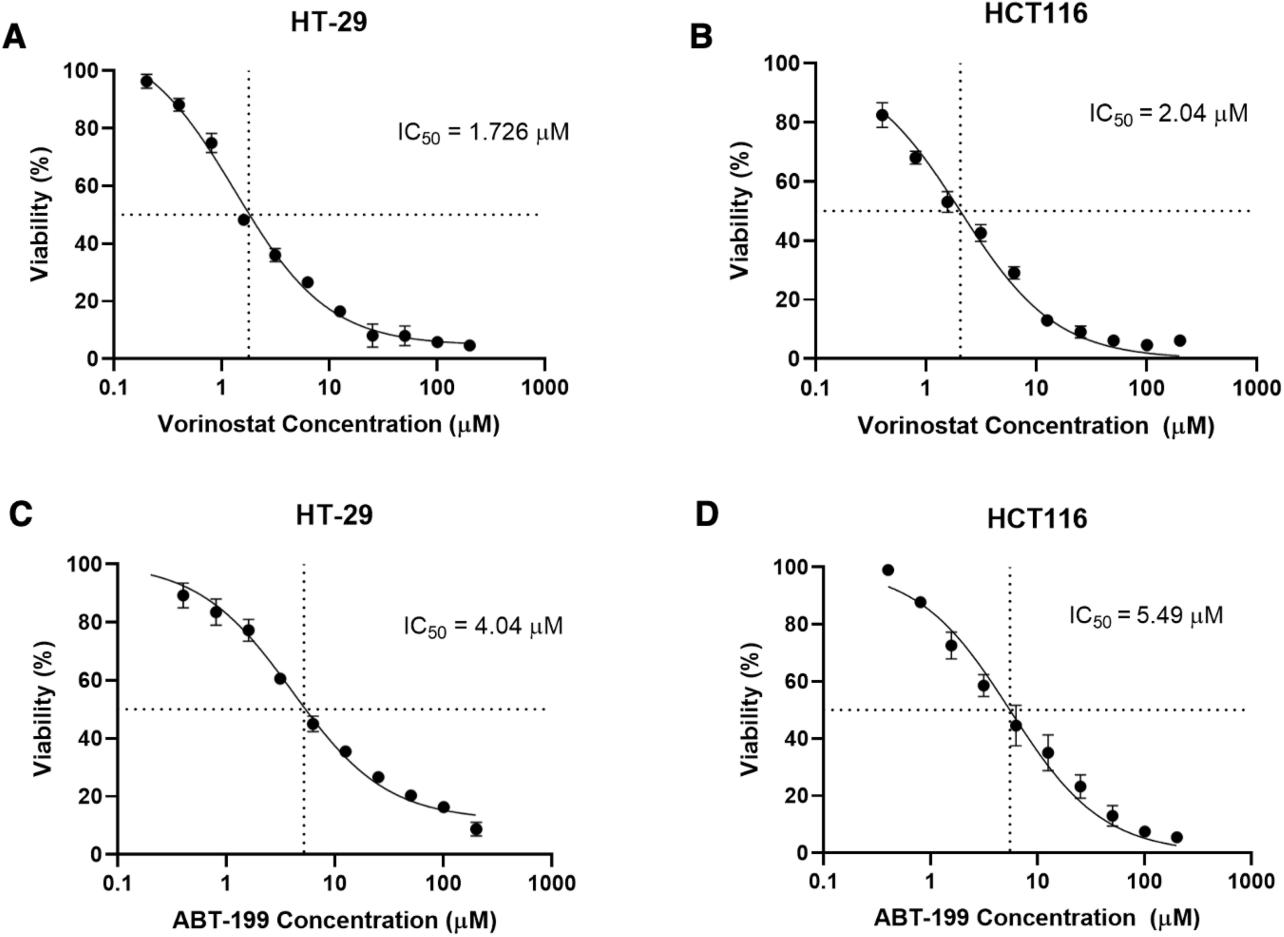
Assessment of Anti-Proliferative Effect of Vorinostat and ABT-199 in HCT116 and HT-29 colorectal cancer cells. HT-29 cells and HCT-116 cells were treated with various concentrations of Vorinostat or ABT-199 for 72 hours prior to cell viability assessment by MTT assay. Cell viability percentage are presented relative to the untreated control. Data represents mean ± SD of three independent experiments.

To establish the baseline sensitivity of colorectal cancer cells to the individual drugs, we assessed the anti-proliferative effects of ABT-199 and Vorinostat in HT-29 and HCT116 cell lines. As illustrated in **Fig 1**, both agents significantly inhibited cell viability in a dose-dependent manner, although the degree of sensitivity varied between cell lines.. Vorinostat demonstrated a more pronounced anti-proliferative effect, with an IC_50_ of 1.32 µM in HT-29 cells and 2.04 µM in HCT116 cells at the 72-hours. Similarly, ABT-199 exhibited anti-proliferative activity, with an IC_50_ of 4.04 µM in HT-29 cells and 5.49 µM in HCT116 cells at 72 hours.

### 3.2. Investigating the nature of interaction between Vorinostat and ABT-199 in colorectal cancer cells

**Fig 2:**
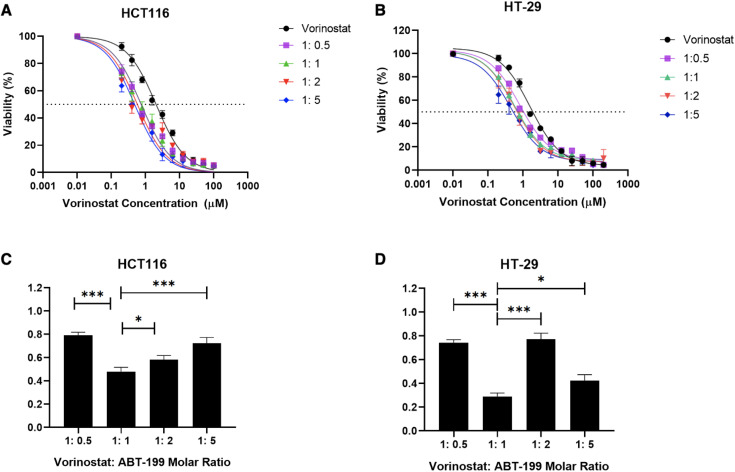
Dose-response effects and combination index (CI) analysis of Vorinostat and ABT-199 combination in colorectal cancer cells. HT-29 **(A)** and HCT116 **(B)** cells were treated with various molar ratios of Vorinostat: ABT-199 for 72 hours, and cell viability was measured using the MTT assay. CI values for the Vorinostat and ABT-199 co-treatment were calculated in HT-29 **(C)** and HCT116 **(D)** at fraction of affected (Fa) at 0.9 (i.e., Fa of 0.9 indicates 90% cell death or growth inhibition). Data represents the mean ± SD of three independent experiments.

The subsequent phase of the study focused on exploring the interaction between Vorinostat and ABT-199. The primary aim was to determine whether the co-administration of the two agents results in a synergistic effect against colorectal cancer cells and to identify the optimal drug combination ratio that maximized this synergistic effect. To achieve this, HT-29 and HCT116 cells were treated with varying molar ratios of Vorinostat and ABT-199 (1:0.5, 1:1, 1:2, and 1:5) for 72 hours, and cell viability was subsequently assessed using the MTT assay, as presented in **Fig 2A and 2B. To quantify the nature of the drug interactions,** The combination index (CI) was calculated according to the Chou-Talalay method [15] using CompuSyn. As shown in **Fig 2C and 2D**, all tested combinations produced CI values below 1, indicating synergistic effect in both cell lines. Among the tested ratios, the 1:1 molar ratio consistently demonstrated the strongest synergism, exhibiting the lowest CI values across both HT-29 and HCT116 cells. Importantly, statistical analysis revealed that the 1:1 molar ratio exhibited significantly lower CI values compared to the other tested ratios, indicating that this ratio resulted in the strongest synergistic interaction.

### 3.3. Preparation and characterization of ABT-199- and Vorinostat-Loaded within PLGA nanoparticles

Given the poor solubility and limited bioavailability of both ABT-199 and Vorinostat, encapsulation in PLGA NPs was pursued to improve their therapeutic potential. Encapsulation improves the stability and controlled release of these agents, potentially increasing their efficacy while reducing off-target effects. Additionally, co-encapsulation allows for precise control over drug ratios, ensuring the synergistic effects observed in colorectal cancer cells are maintained.

Following confirmation of synergistic effects against colorectal cancer cells, four nanoparticle formulations were prepared: ABT-199-loaded nanoparticles (ABT-NPs), Vorinostat-loaded nanoparticles (VOR-NPs), dual-loaded PLGA nanoparticles (DLNPs), and Blank PLGA nanoparticles (BNPs) as controls.

Following synthesis, the nanoparticles were characterized for their physicochemical properties, including size, PDI, and zeta potential, as well as EE%. The characterization data, presented in **Table 1**, revealed that the BNPs had an average diameter of 137.5 ± 7.4 nm, with a PDI of 0.087 ± 0.03, indicating a highly uniform particle distribution. The zeta potential of BNPs was –23.4 ± 4.6 mV, suggesting good stability due to the negative surface charge.

**Table 1:** Physicochemical Characterization of BNPs, ABT-NPs, VOR-NPs and DLNPs.

Parameter	Diameter (nm)	PDI	Zeta potential (mv)	EE% (ABT-199)	EE% (Vorinostat)	Molar ratio
BNPs	137.5 ± 7.4	0.087 ± 0.03	–23.4 ± −4.6	–	–	–
ABT-NPs	210.6 ± 6.2	0.154 ± 0.04	–20.4 ± −2.1	73.2 ± 4.81	–	–
VOR-NPs	202.5 ± 5.6	0.104 ± 0.03	–20.4 ± −2.1	–	86.4 ± 5.5	–
DLNPs	210 ± 7.3	0.149 ± 0.024	−17.3 ± 5.6	64.1 ± 5.2*	65.3 ± 4.8**	1: 2

* p < 0.05 and ** p < 0.01 vs. corresponding single-loaded nanoparticle group (ABT-NPs or VOR-NPs), determined by one-way ANOVA with Tukey’s post-hoc test.

The ABT-NPs displayed a larger average size of 210.6 ± 6.2 nm, likely due to the encapsulation of ABT-199. The PDI for ABT-NPs was 0.154 ± 0.04, indicating a slightly broader size distribution compared to BNPs, though still within acceptable limits for nanoparticle formulations. The zeta potential was –20.4 ± 2.1 mV, reflecting good stability. The encapsulation efficiency (EE) for ABT-199 was determined to be 73.2 ± 4.81%, demonstrating successful drug loading.

Similarly, the VOR-NPs had an average size of 202.5 ± 5.6 nm, with a PDI of 0.104 ± 0.03, indicating good particle uniformity. The zeta potential was consistent with that of ABT-NPs at –20.4 ± 2.1 mV. Vorinostat’s encapsulation efficiency was higher, measured at 86.4 ± 5.5%, indicating effective drug incorporation within the nanoparticles. The DLNPs, which co-encapsulated ABT-199 and Vorinostat, had an average size of 210 ± 7.3 nm and a PDI of 0.149 ± 0.024, demonstrating uniform size distribution similar to the single-loaded formulations. The zeta potential for DLNPs was slightly lower, recorded at –17.3 ± 5.6 mV. The concentration of ABT-199 within DLNPs was measured at 115 ± 26.42 nm, while the concentration of Vorinostat was 330 ± 46.2 nm, maintaining a molar ratio of 1:2, which aligns with the synergistic ratio identified in earlier cytotoxicity experiments.

To complement the DLS data, the intensity-based size distribution profiles for all nanoparticle formulations are provided in S2 Fig, further confirming monodispersity and the absence of aggregation.

**Fig 3:**
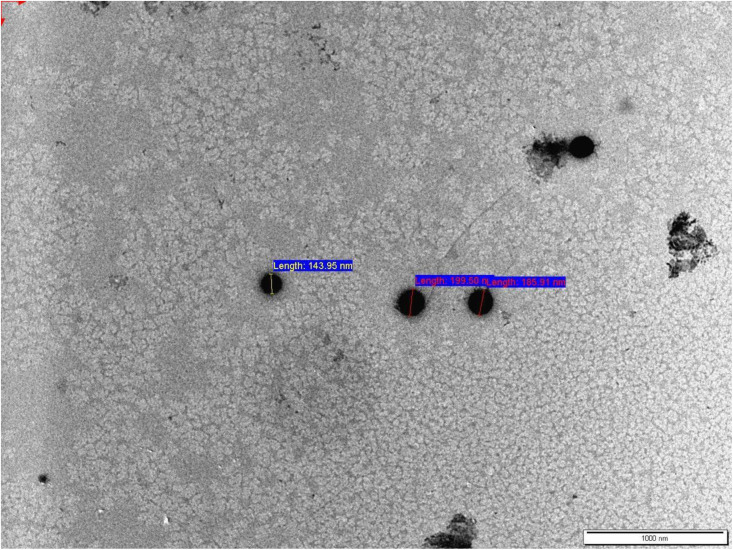
TEM image of DLNPs The nanoparticles appear spherical in shape with smooth surfaces and an average size of approximately 200 nm. Scale bar = 1000 nm.

To further assess nanoparticle morphology, TEM analysis was performed on the DLNP formulation. As shown in **Fig 3**, the particles appeared spherical in shape, with a diameter consistent with dynamic light scattering results, approximately 200 nm. The images confirmed the uniform size distribution and structural integrity of the nanoparticles.

**Fig 4:**
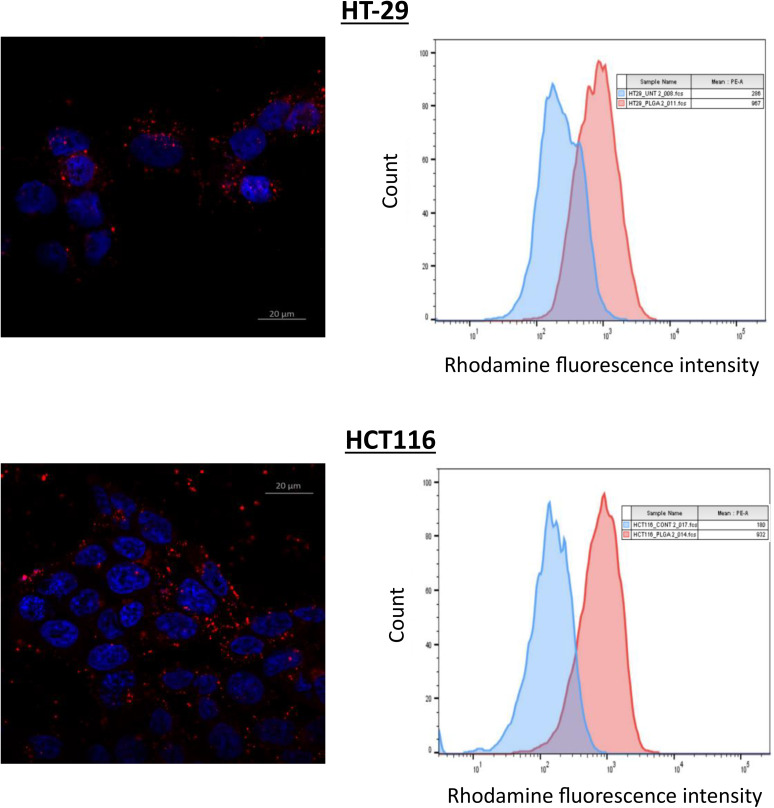
Assessment of nanoparticle uptake in colorectal cancer cells using confocal microscopy and flow cytometry. HT-29 and HCT116 cells were treated with 200 µg/mL of Rhodamine B-loaded nanoparticles for 6 hours. Following treatment, nanoparticle uptake was evaluated through flow cytometry and confocal microscopy analyses to visualize and quantify the internalization of Rhodamine B-labeled nanoparticles within the cells.

To validate the intracellular delivery capability of the nanoparticles, an uptake study was conducted using a traceable Rhodamine B-conjugated PLGA nanoparticles. Following 6 hours of incubation, both flow cytometry and confocal microscopy revealed substantial internalization of the nanoparticles, as indicated by a significant increase in rhodamine fluorescence intensity in treated cells compared to untreated controls (**Fig 4**), These findings confirm efficient cellular uptake of the nanoparticles, supporting their potential for effective intracellular drug delivery.

Furthermore, the nanoparticles exhibited robust physicochemical stability under various storage conditions, with minimal fluctuations in size, polydispersity index (PDI), and zeta potential over time (S3 Fig).

**Fig 5:**
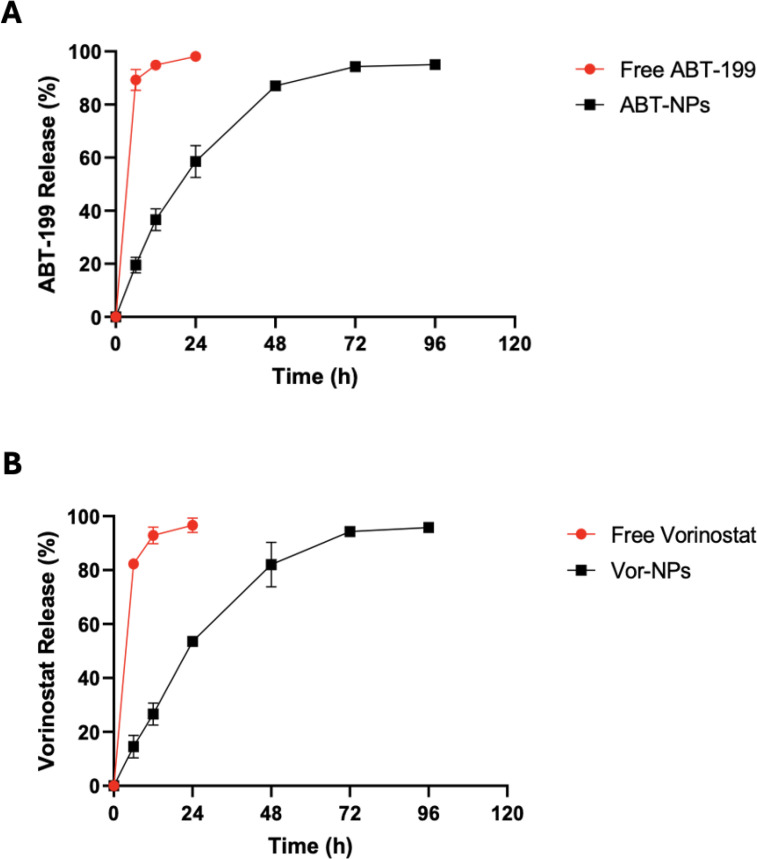
Drug release kinetics of ABT-199 (A) and Vorinostat (B). A total amount of 10 mg of each NP formulation was re-suspended in 3 ml of PBS and injected in a dialysis bag which was then immersed in a PBS containing 10% FBS. At the indicated time points, the remaining NP pellet was removed and quantified. Data represent mean ± Standard Error of the Mean (SEM).

In addition to the physicochemical properties, the *in vitro* release kinetics of ABT-199 and Vorinostat from the PLGA nanoparticles were evaluated. ABT-199 exhibited a biphasic release profile, characterized by an initial burst release of 58% within the first 24 hours, followed by a sustained release over the subsequent 72 hours. In contrast, free ABT-199 control demonstrated a complete release within 6 hours (**Fig 5A**). Vorinostat followed a similar biphasic release pattern, with 56.76% released within the first 24 hours, followed by a slower release over the next 72 hours, wherease the free Vorinostat control also showed complete release within 6 hours (**Fig 5B**). The initial burst release observed in both formulations is likely due to the fraction of drug adsorbed or weakly associated with the nanoparticle surface, while the subsequent sustained phase reflects the controlled diffusion of the drug encapsulated within the PLGA matrix.

### 3.4. Evaluating the antiproliferative effect of ABT-NPs, VOR-NPs and DLNPs on colorectal cancer cell lines

To determine whether nanoencapsulation affected the anti-proliferative activity of ABT-199 and Vorinostat, we compared the the anti-proliferative effects of ABT-NPs and VOR-NPs were with their free forms in colorectal cancer. Prior to this, it was crucial to confirm that the BNPs exhibited no inherent cytotoxicity. Therefore, HT-29 and HCT116 cells were treated with escalating concentrations of BNPs for 72 hours, and cell viability was assessed using the MTT assay. As shown in S4 Fig, BNPs did not induce any significant reduction in cell viability at any tested concentration, confirming their biocompatibility and validating that subsequent cytotoxic effects were attributable solely to the encapsulated active compounds.

**Fig 6:**
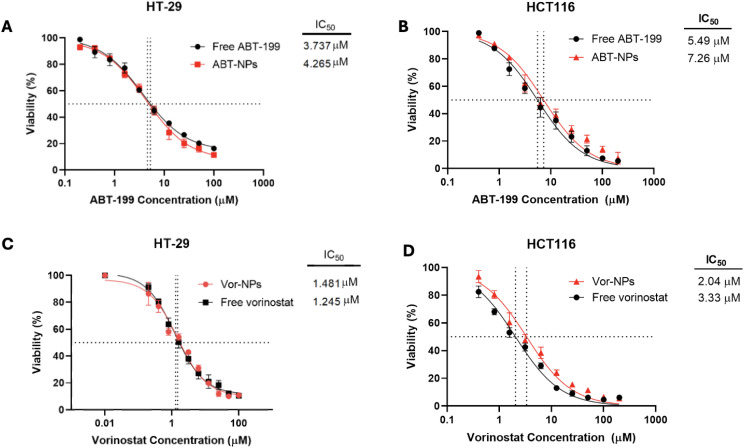
Comparative cytotoxicity of free and nanoparticle-encapsulated ABT-199 and Vorinostat in colorectal cancer cells. **(A, B)** Dose–response curves of free ABT-199 versus ABT-199-loaded nanoparticles (ABT-NPs) in HT-29 and HCT116 cells, respectively, after 72-hour treatment. **(C, D)** Dose–response curves of free Vorinostat versus Vorinostat-loaded nanoparticles (VOR-NPs) in HT-29 and HCT116 cells, respectively. Cell viability percentage are presented relative to the untreated control. Data represents mean ± SD of three independent experiments.

Regarding ABT-NPs, ABT-NPs preserved a cytotoxic effect comparable to the free drug. In HT-29 cells, the IC₅₀ value for ABT-NPs was 4.27 µM versus 3.74 µM for free ABT-199, while in HCT116 cells, the IC₅₀ values were 7.26 µM and 5.49 µM, respectively (**Fig 6A and 6B**). Although a modest shift in potency was observed, statistical analysis confirmed that this difference was not significant, indicating that encapsulation did not compromise drug efficacy.

Similarly, Vorinostat-loaded nanoparticles (VOR-NPs) retained potent antiproliferative activity. The IC₅₀ values for VOR-NPs in HT-29 and HCT116 cells were 1.48 µM and 3.33 µM, respectively, compared to 1.25 µM and 2.04 µM for the free drug (**Fig 6C and 6D**). Again, while slight increases in IC₅₀ values were noted, these were not statistically significant, underscoring that the nanoformulation maintained the therapeutic potential of Vorinostat.

Collectively, these findings confirm that nanoencapsulation using PLGA did not hinder the cytotoxic activity of either ABT-199 or Vorinostat. Instead, it offers a promising delivery strategy with potential advantages in drug solubility, stability, and co-delivery for future in vivo applications.

### 3.5. Apoptosis Induction by the nanoparticle formulations

To determine whether nanoencapsulation of ABT-199 and Vorinostat enhances apoptosis in colorectal cancer cells, we evaluated early and late apoptotic cell populations using Annexin V-FITC/PI staining followed by flow cytometric analysis in HCT116 and HT-29 cell lines.

**Fig 7:**
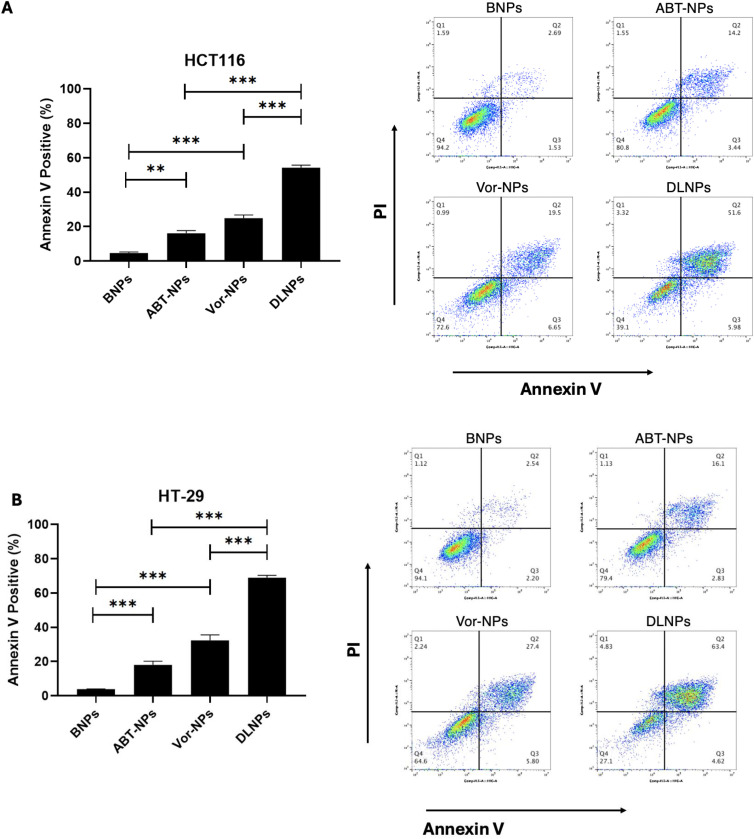
Apoptosis analysis of colorectal cancer cells following nanoparticle treatment using Annexin V/PI staining. **(A)** HCT116 and **(B)** HT-29 cells were treated with BNPs, 2 µM ABT-NPs, 1 µM VOR-NPs, or equivalent concentration of DLNPs for 72 hours. Apoptosis was assessed using Annexin V-FITC/PI staining and quantified by flow cytometry. The bar graphs show the percentage of Annexin V-positive cells (early and late apoptotic), while representative dot plots depict cell distribution across quadrants (Q1 = necrotic, Q2 = late apoptotic, Q3 = early apoptotic, Q4 = viable). Data are presented as mean ± SD (n = 3). Statistical significance was determined by one-way ANOVA followed by Tukey’s post hoc test. * *p* < 0.05, ** *p* < 0.01, *** *p* < 0.001.

In HCT116 cells, treatment with BNPs resulted in minimal apoptosis (Annexin V ⁺ : 4.2%). In contrast, treatment with ABT-NPs and VOR-NPs increased the percentage of Annexin V-positive cells to 17.6% and 26.15%, respectively. Notably, cells treated with DLNPs showed a dramatic increase in apoptosis, with 57.58% Annexin V-positive cells (Fig 7A). Statistical analysis confirmed that DLNPs induced significantly higher apoptosis compared to single-drug-loaded NPs and BNPs (p < 0.001).

A similar trend was observed in HT-29 cells (Fig 7B). BNPs showed negligible apoptotic effect (4.74% Annexin V-positive), while ABT-NPs and VOR-NPs induced 18.9% and 33.2% Annexin V-positive cells, respectively. The DLNP-treated group exhibited a marked apoptotic response, with 68.02% Annexin V-positive cells, significantly exceeding all other groups (p < 0.001).

Representative quadrant dot plots from flow cytometry further supported these findings, showing increased populations in the upper right quadrant (Annexin V ⁺ /PI⁺) and lower right quadrant (Annexin V ⁺ /PI⁻) in cells treated with DLNPs, consistent with a mix of late and early apoptosis, respectively.

To further confirm the apoptotic mechanism underlying the enhanced cytotoxicity of the dual-loaded nanoparticles, caspase-3 activity—a key executioner protease in apoptosis—was measured in HT-29 and HCT116 cells following treatment with BNPs, ABT-NPs, VOR-NPs, or DLNPs.

**Fig 8:**
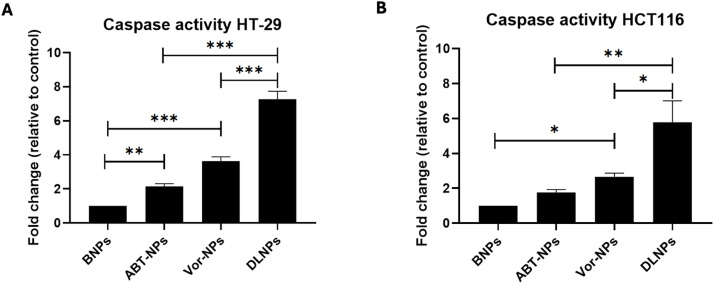
Induction of caspase-3 activity in HT-29 and HCT116 cells treated with ABT-NPs, VOR-NPs, or DLNPs. (A) HT-29 and (B) HCT116 cells were treated with BNPs, 2 µM ABT-NPs, 1 µM VOR-NPs, or equivalent concentration of DLNPs for 72 hours Caspase-3 activity was quantified using a colorimetric caspase-3 assay and expressed as fold change relative to BNPs controls. Data are shown as mean ± SD (n = 3). Statistical significance was determined using one-way ANOVA with Tukey’s post hoc test. * *p* < 0.05, ** *p* < 0.01, *** *p* < 0.001.

In HT-29 cells, caspase-3 activity was significantly elevated in response to all treatment groups compared to the blank nanoparticles (p < 0.01 for ABT-NPs; p < 0.001 for VOR-NPs and DLNPs). Notably, DLNPs triggered the highest fold change in caspase-3 activity (8.2-fold relative to control), significantly exceeding the activity observed with either ABT-NPs or VOR-NPs alone (p < 0.001; Fig 8A).

In HCT116 cells, a similar trend was observed. While ABT-NPs and VOR-NPs induced modest increases in caspase-3 activation (1.8-fold and 2.6-fold, respectively), DLNPs induced a 5.8-fold increase relative to BNPs (p < 0.01). The difference in caspase-3 activity between DLNPs and each single-drug nanoparticle formulation was statistically significant (p < 0.05 and p < 0.01, respectively; Fig 8B).

These results reinforce that co-delivery of ABT-199 and Vorinostat via PLGA nanoparticles significantly enhances apoptosis through activation of the caspase-3 cascade, further supporting the superior pro-apoptotic efficacy of the dual-loaded formulation.

## 4. Discussion

This study presents the development and evaluation of PLGA NPs co-encapsulating ABT-199 (Venetoclax), a selective B-cell lymphoma-2 (BCL-2) inhibitor, and Vorinostat, a pan-histone deacetylase (HDAC) inhibitor, to achieve synergistic cytotoxicity in colorectal cancer cell lines HT-29 and HCT116. Our findings demonstrate that these DLNPs significantly enhance pro-apoptotic and cytotoxic effects compared to single-agent treatments or free drug combinations, as evidenced by increased Annexin V-positive cell populations, elevated cleaved caspase-3 levels, and pronounced DNA fragmentation. The observed synergy arises from the mechanistic interplay between BCL-2 inhibition and HDAC inhibition, which targets complementary survival pathways in cancer cells. By leveraging the PLGA nanoparticle platform, we overcome critical limitations of free ABT-199 and Vorinostat, including poor pharmacokinetics, dose-limiting toxicities, and variable tumor deposition, thereby enhancing therapeutic efficacy and paving the way for clinical translation.

The synergistic cytotoxicity of ABT-199 and Vorinostat, delivered via DLNPs, is rooted in their complementary effects on intrinsic and extrinsic apoptosis pathways. ABT-199, a BH3-mimetic, selectively binds to BCL-2, a key anti-apoptotic protein in the intrinsic (mitochondrial) apoptosis pathway, preventing it from sequestering pro-apoptotic BH3-only proteins such as Bim and Bad [16]. This interaction promotes mitochondrial outer membrane permeabilization (MOMP), releasing cytochrome c and activating executioner caspases like caspase-3 and −7, which drive programmed cell death [17]. However, colorectal cancer cells frequently exhibit resistance to BCL-2 inhibition due to compensatory upregulation of other anti-apoptotic BCL-2 family members, notably MCL-1, which is overexpressed in colorectal cancer and mediates drug resistance [18–20]. Vorinostat, as a pan-HDAC inhibitor, counters this resistance by modulating epigenetic and non-epigenetic processes. HDAC inhibition leads to hyperacetylation of histones and non-histone proteins, reactivating silenced tumor suppressor genes and pro-apoptotic factors (e.g., Bim/BCL2L11, TRAIL receptors, p21) while downregulating anti-apoptotic proteins such as MCL-1, BCL-xL, and survivin [21–23]. This dual targeting creates a pro-apoptotic environment where Vorinostat lowers the apoptotic threshold by upregulating BH3-only proteins and death receptors, while ABT-199 ensures BCL-2 cannot neutralize these signals, resulting in robust MOMP and caspase activation.

Our apoptosis assays corroborated this synergy, revealing a significantly higher fraction of early and late apoptotic cells in DLNP-treated cultures compared to monotherapies. Flow cytometry data showed that DLNPs induced synergism in HT-29 and HCT116 cells across various dose ranges, consistent with prior studies demonstrating synergistic cancer cell death with BCL-2/HDAC inhibitor combinations [24–27].

The PLGA nanoparticle platform plays a pivotal role in translating this mechanistic synergy into therapeutic efficacy. The DLNPs, with a hydrodynamic diameter of 200 nm and a low PDI (< 0.2), are optimally sized for tumor accumulation via the enhanced permeability and retention (EPR) effect, exploiting the leaky vasculature and impaired lymphatic drainage of solid tumors. Their moderately negative zeta potential ensures colloidal stability in biological media while facilitating cellular uptake through receptor-independent endocytosis. Confocal microscopy and flow cytometry experiments confirmed robust internalization of fluorescently labeled NPs in HT-29 and HCT116 cells, with significantly high intracellular fluorescence intensity. Quantitative flow cytometry revealed a 2–3-fold increase in drug accumulation with NPs, aligning with literature reports of enhanced cellular uptake with polymeric nanocarriers [28–31].

The DLNPs’ ability to co-deliver ABT-199 and Vorinostat at a synergistic molar ratio is a critical advantage over free drug combinations, which suffer from disparate uptake kinetics and pharmacokinetic profiles [14]. Free ABT-199 and Vorinostat exhibit rapid clearance and metabolism, leading to suboptimal drug ratios in tumor cells. In contrast, DLNPs ensure synchronized delivery, with each internalized nanoparticle releasing both drugs intracellularly, maintaining the synergistic ratio throughout the therapeutic window [32]. Confocal imaging further revealed cytosolic localization of DLNPs, indicating successful endosomal escape, likely facilitated by PLGA’s proton sponge effect or lipid membrane interactions. This co-localized delivery enhances the uniformity of apoptosis across cell populations, as evidenced by consistent Annexin V/PI staining patterns in treated cultures.

The DLNPs’ biphasic drug release profile—initial burst followed by sustained release over 2–3 days—optimizes therapeutic efficacy. The burst phase, driven by diffusion of surface-associated drug molecules, rapidly achieves cytotoxic concentrations, inducing early apoptosis in a subset of cancer cells within hours. The sustained phase, resulting from gradual PLGA hydrolysis, ensures prolonged drug exposure, targeting slower-proliferating or less accessible tumor cells, such as those in hypoxic or quiescent niches [33]. *In vitro* release studies showed that both ABT-199 and Vorinostat exhibited similar release kinetics, maintaining the 1:2 molar ratio throughout the study duration, which is critical for sustaining synergistic interactions.

The tumor microenvironment (TME) in colorectal cancer, characterized by abnormal vasculature, hypoxia, acidity (pH ~ 6.5–6.8), and dense extracellular matrix, poses significant barriers to drug delivery [34]. The NPs address these challenges by exploiting the EPR effect for passive tumor targeting, with their nanoscale size enabling extravasation through leaky tumor vessels [35]. The slightly acidic TME accelerates PLGA degradation into lactic and glycolic acid monomers, enhancing localized drug release and creating a tumor-specific depot of chemotherapy. This environment-responsive release minimizes off-target exposure, potentially reducing systemic toxicities such as Vorinostat’s hematologic side effects (thrombocytopenia) or ABT-199’s on-target platelet toxicity [36]. Moreover, nanoparticle encapsulation shields drugs from premature degradation or efflux by multidrug resistance pumps (e.g., P-glycoprotein), ensuring higher intratumoral concentrations compared to free drugs, as supported by studies of PLGA-based nanocarriers [37].

Our findings build on a growing body of evidence supporting nanoformulations for combination cancer therapy. Zhang et al. demonstrated that co-encapsulated etoposide and cisplatin in polymeric NPs achieved significant tumor growth inhibition in non-small cell lung cancer models, surpassing free drug combinations [38]. Similarly, Abed et al. reported enhanced apoptosis and reduced leukopenia with co-delivered RG7388 and Entinostat in p53 wild-type colorectal cancer cells, highlighting nanotechnology’s ability to mitigate dose-limiting toxicities [39]. Our DLNPs achieved comparable efficacy to free drugs at potentially lower doses, suggesting a wider therapeutic window. The clinical relevance of this approach is underscored by FDA-approved nanoformulations like Vyxeos® (daunorubicin and cytarabine) for acute myeloid leukemia and Lipoxal^TM^ (irinotecan and floxuridine), which advanced to phase II trials for advanced colorectal cancer [40,41]. These precedents validate co-delivery strategies for optimizing drug ratios, enhancing tumor-specific delivery, and reducing systemic toxicity.

## 5. Conclusion and future perspectives

This study demonstrates the successful development of PLGA-based nanoparticles co-loaded with ABT-199 and Vorinostat, achieving a controlled 1:2 molar ratio that maintained strong synergistic anticancer activity in colorectal cancer cells. The nanoformulations exhibited favorable physicochemical characteristics, efficient cellular uptake, sustained drug release, and enhanced apoptotic induction compared to free drugs. Importantly, co-encapsulation allowed synchronized drug delivery, potentially overcoming pharmacokinetic disparities and resistance mechanisms.

While our in vitro results are promising, several limitations warrant consideration. The HT-29 and HCT116 cell lines, while representative of colorectal cancer, do not fully recapitulate the heterogeneity of primary tumors or the TME’s complexity. *In vivo* studies are essential to evaluate the DLNPs’ tumor-targeting efficiency, biodistribution, and safety profile, particularly in orthotopic or patient-derived xenograft models. Pharmacokinetic studies will clarify whether the DLNPs achieve sustained drug levels in tumors while minimizing systemic exposure. Additionally, the potential for immune modulation by Vorinostat suggests synergy with checkpoint inhibitors or adoptive cell therapies, which could be explored in immunocompetent models. Long-term toxicity studies are also needed to assess the impact of repeated DLNP dosing, given the known hematologic and gastrointestinal toxicities of free ABT-199 and Vorinostat [17,30].

Future research will focus on optimizing DLNP formulations, such as incorporating active targeting ligands (e.g., anti-EGFR antibodies) to enhance tumor specificity or adjusting PLGA composition to fine-tune release kinetics. The versatility of the PLGA platform allows extension to other synergistic drug pairs, tailored to the molecular vulnerabilities of different tumor types (e.g., BCL-xL inhibitors for pancreatic cancer or PARP inhibitors for BRCA-mutant cancers). Exploring the DLNPs’ impact on 3D tumor spheroids or organoids could bridge the gap between *in vitro* and *in vivo* models, providing insights into penetration and efficacy in complex tumor architectures.

## Supporting information

S1 Fig FTIR Spectrum of DMSO and Blank PLGA NPs. (DOCX)

S2 Fig Intensity-based DLS size distribution profiles of PLGA nanoparticle formulations. Representative DLS data displaying the hydrodynamic size distribution (by intensity) for BNPs (A), ABT-NPs (B), VOR-NPs (C), and DLNPs (D). All formulations exhibited unimodal distributions with narrow peaks, indicating uniform particle populations and absence of aggregation.
(DOCX)

S3 Fig Assessment of PLGA BNPs stability in terms of size (A), PDI (B), and zeta potential (C). Data presented as mean ± SEM.
(DOCX)

S4 Fig Assessment of the cytotoxicity of BNPs. HT-29 cells were treated with varying concentrations of BNPs for 72-hours prior to MTT viability assay analysis. Data represent the mean ± SD of three independent experiments.
(DOCX)
