# Supplementary figures and images for: Synergistic anticancer effects of ABT-199 and Vorinostat encapsulated in PLGA nanoparticles: Formulation, characterization, and antiproliferative effects against colorectal cancer cells
Source: PLoS One. 2025 Oct 10;20(10):e0334427. doi: 10.1371/journal.pone.0334427 (PMC12513621; doi:10.1371/journal.pone.0334427)

**
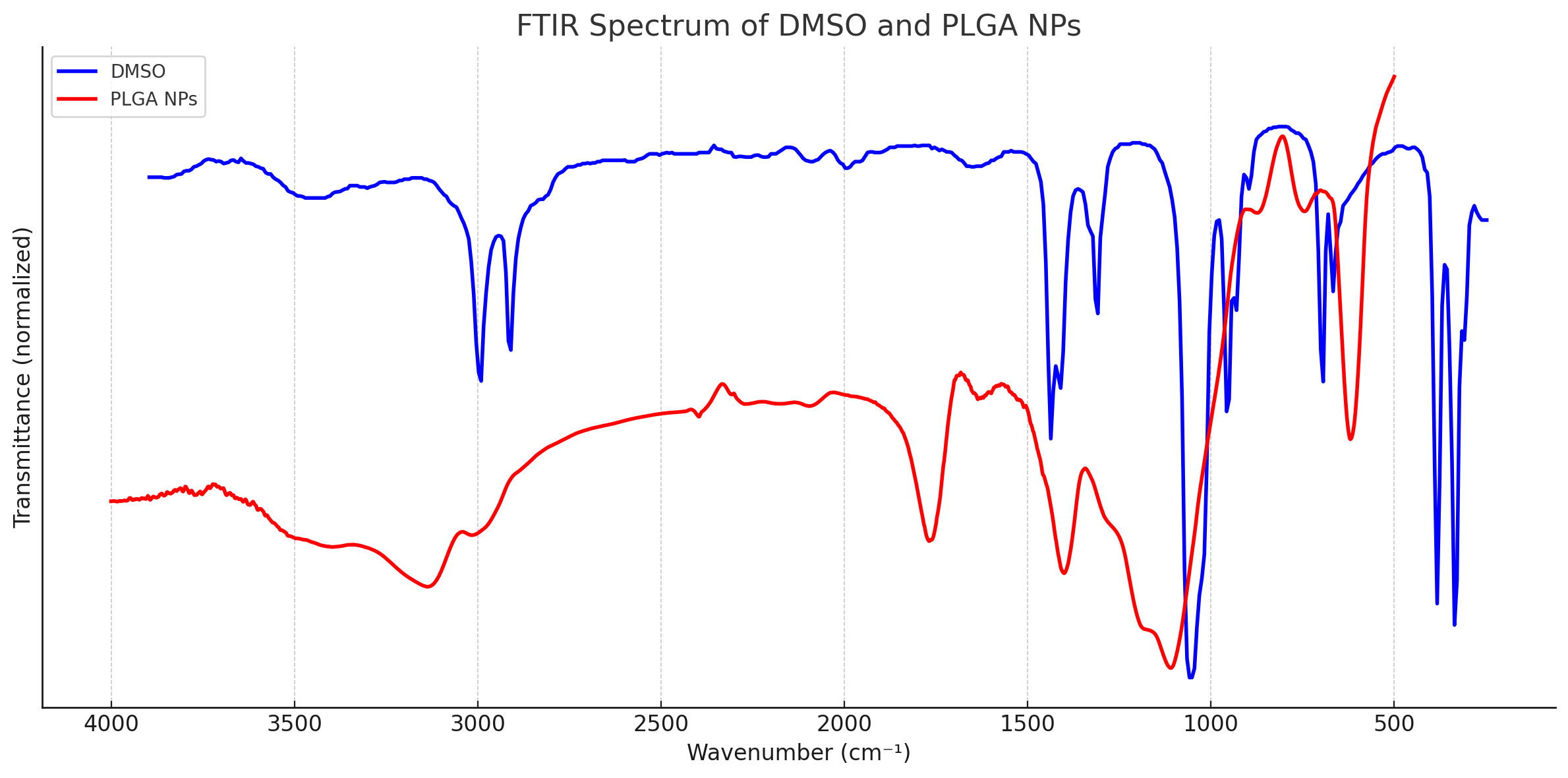
**

**Supplementary Figure 1. FTIR Spectrum of DMSO and Blank PLGA NPs**

Supplement: S1 Fig — (DOCX) [file pone.0334427.s001.docx]
