# Supplementary material for: Synergistic anticancer effects of ABT-199 and Vorinostat encapsulated in PLGA nanoparticles: Formulation, characterization, and antiproliferative effects against colorectal cancer cells
Source: PLoS One. 2025 Oct 10;20(10):e0334427. doi: 10.1371/journal.pone.0334427 (PMC12513621; doi:10.1371/journal.pone.0334427)
Supplement: S2 Fig — Representative DLS data displaying the hydrodynamic size distribution (by intensity) for BNPs (A), ABT-NPs (B), VOR-NPs (C), and DLNPs (D). All formulations exhibited unimodal distributions with narrow peaks, indicating uniform particle populations and absence of aggregation. (DOCX) [file pone.0334427.s002.docx]

**
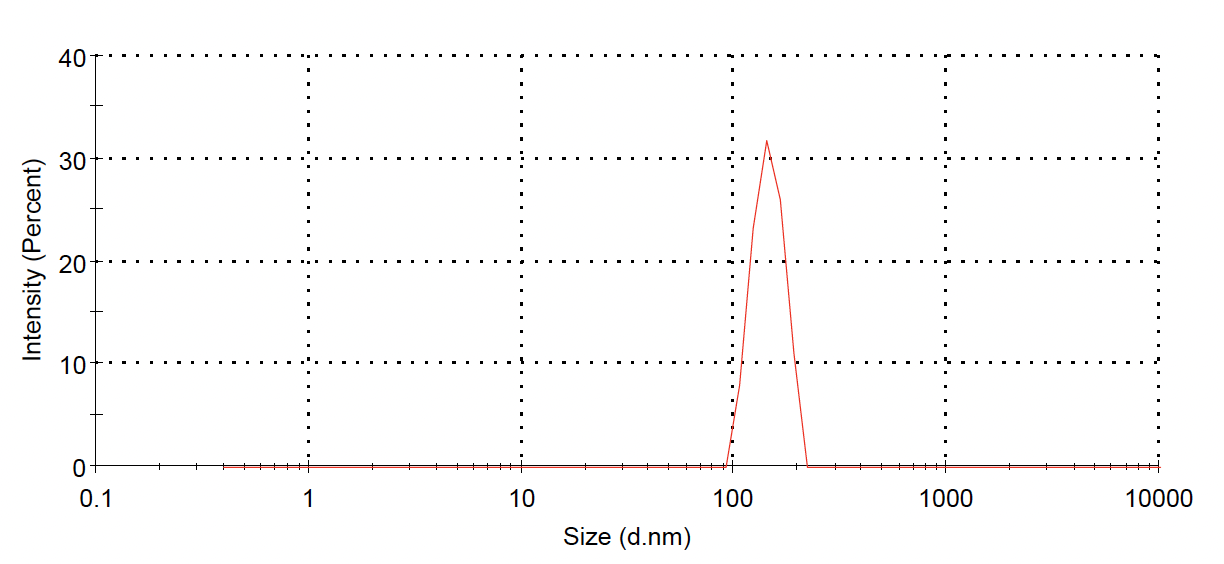

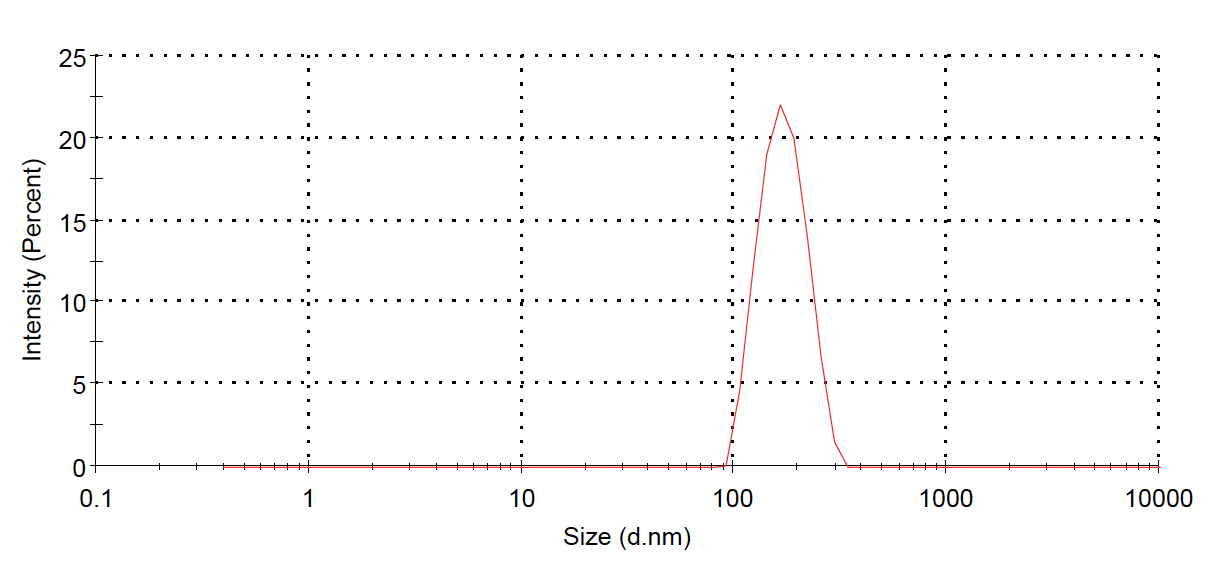

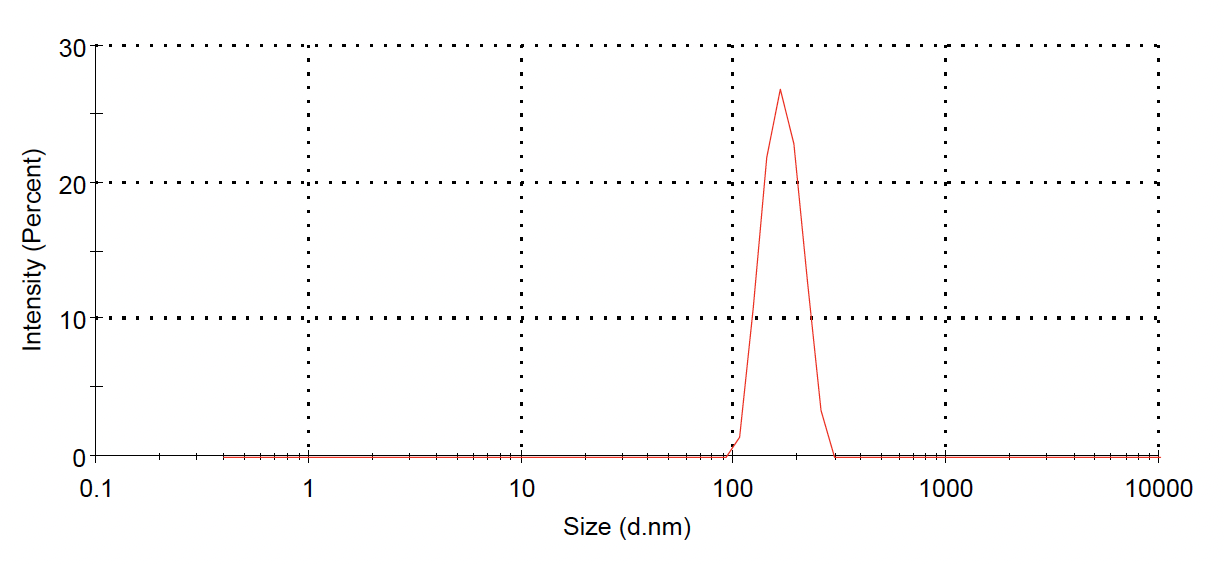
**

**Vor-NPs**

**ABT-NPs**

**BNPs**

**D**

**B**

**C**

**A**

**
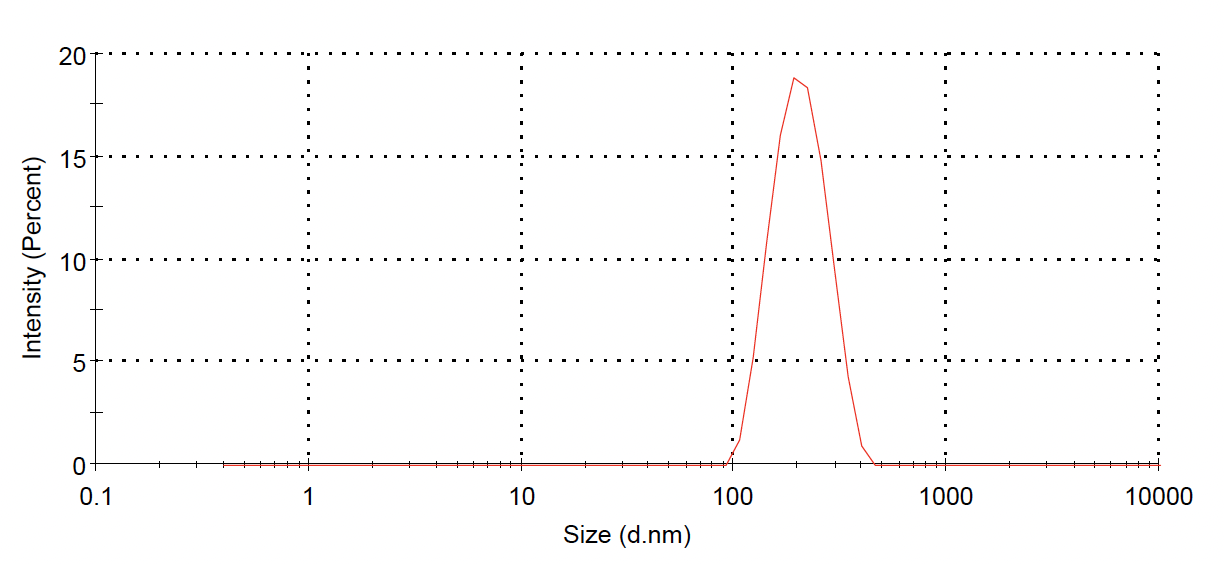
**

**DLNPs**

**Supplementary Figure 2. Intensity-based DLS size distribution profiles of PLGA nanoparticle formulations.** Representative DLS data displaying the hydrodynamic size distribution (by intensity) for BNPs (A), ABT-NPs (B), VOR-NPs (C), and DLNPs (D).
