# Supplementary material for: Synergistic anticancer effects of ABT-199 and Vorinostat encapsulated in PLGA nanoparticles: Formulation, characterization, and antiproliferative effects against colorectal cancer cells
Source: PLoS One. 2025 Oct 10;20(10):e0334427. doi: 10.1371/journal.pone.0334427 (PMC12513621; doi:10.1371/journal.pone.0334427)
Supplement: S3 Fig — Data presented as mean ± SEM. (DOCX) [file pone.0334427.s003.docx]

**A**


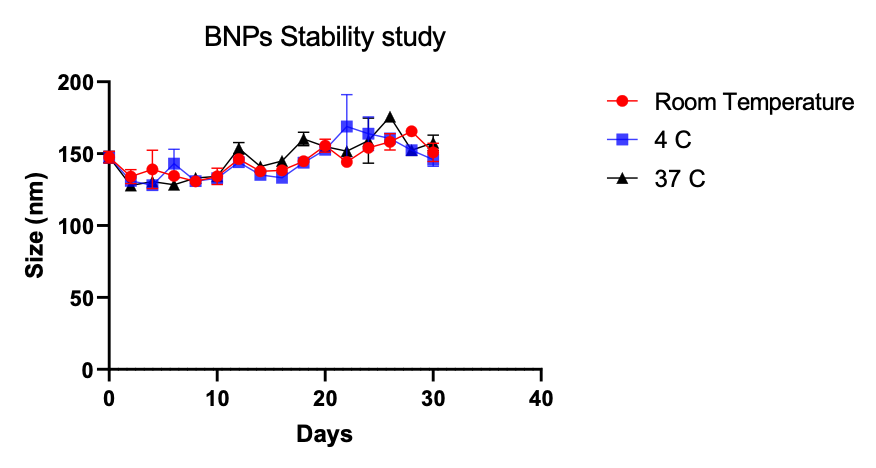


**B**


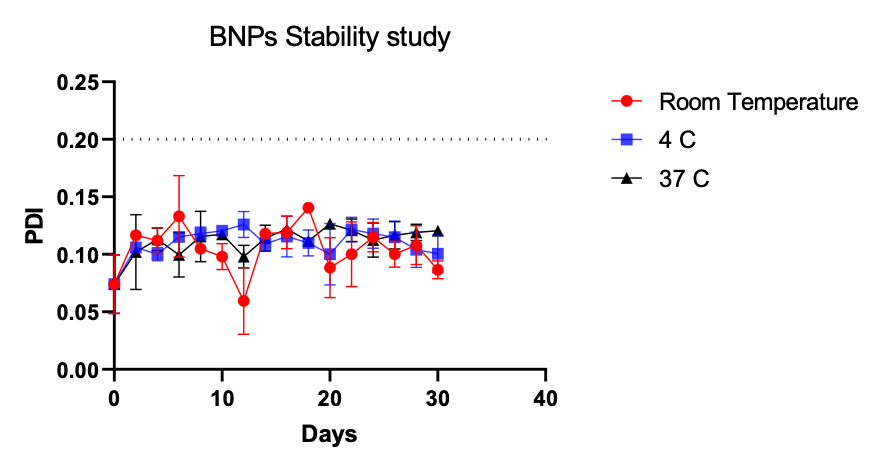


**C**


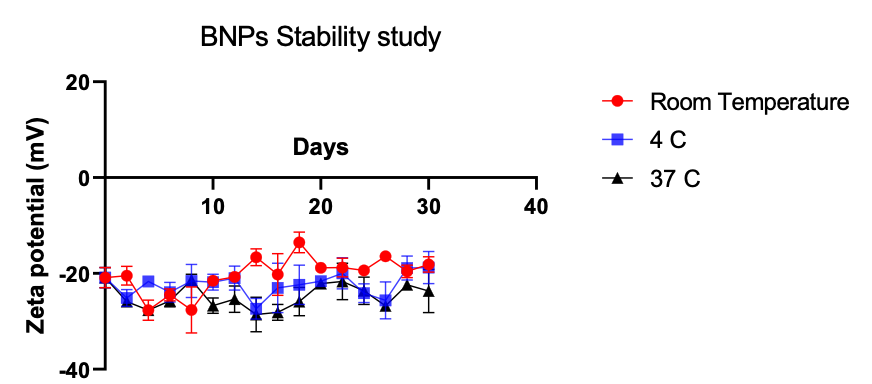


**Supplementary Figure 3. Assessment of PLGA BNPs stability in terms of size (A), PDI (B), and zeta potential (C).** Data presented as mean ± SEM.
