# Supplementary material for: Synergistic anticancer effects of ABT-199 and Vorinostat encapsulated in PLGA nanoparticles: Formulation, characterization, and antiproliferative effects against colorectal cancer cells
Source: PLoS One. 2025 Oct 10;20(10):e0334427. doi: 10.1371/journal.pone.0334427 (PMC12513621; doi:10.1371/journal.pone.0334427)
Supplement: S4 Fig — HT-29 cells were treated with varying concentrations of BNPs for 72-hours prior to MTT viability assay analysis. Data represent the mean ± SD of three independent experiments. (DOCX) [file pone.0334427.s004.docx]

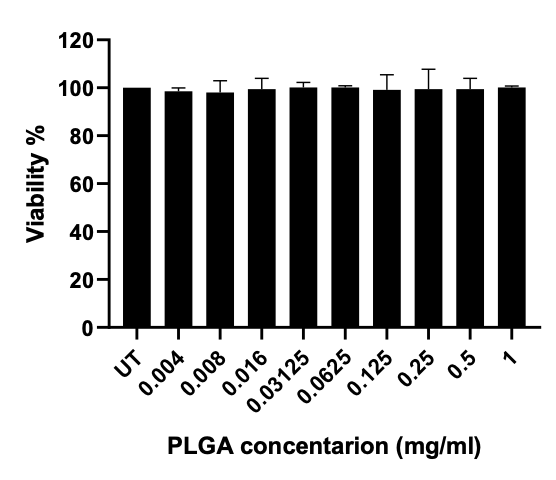


**Supplementary Figure 4. Assessment of the cytotoxicity of BNPs.** HT-29 cells were treated with varying concentrations of BNPs for 72-hours prior to MTT viability assay analysis. Data represent the mean ± SD of three independent experiments.
